# Androgens and Anemia: Current Trends and Future Prospects

**DOI:** 10.3389/fendo.2019.00754

**Published:** 2019-11-14

**Authors:** Ahmed Al-Sharefi, Azmi Mohammed, Altayeb Abdalaziz, Channa N. Jayasena

**Affiliations:** ^1^Section of Investigative Medicine, Imperial College London, Hammersmith Hospital, London, United Kingdom; ^2^The James Cook University Hospital, South Tees Hospitals NHS Foundation Trust, Middlesbrough, United Kingdom; ^3^The Royal Victoria Infirmary, Newcastle Hospitals NHS Foundation Trust, Newcastle upon Tyne, United Kingdom

**Keywords:** anemia, hypogonadism, anemia of chronic kidney disease, testosterone treatment, erythropoesis, haematocrit (HcT)

## Introduction

The regulatory effects of androgens on haematopoiesis have been recognized since the early twentieth century. Castration of male rats causes anemia (1) which is reversible after treatment with androgens (2). A classical clinical study by Vahlquist provided indirect evidence that men had intrinsically higher haematocrit (Hct) levels when compared with women; it was observed that pre-menopausal women did not have higher Hct levels when compared with post-menopausal women, and did have an increased Hct in response to iron supplementation (3). Anecdotal data from competitive athletes suggest that androgen misuse can improve performance, partly through increasing the VO_2_max (Hb-mediated blood oxygen carrying capacity), albeit at the expense of an increased risk of arterial and venous thrombosis (4). Equally, women suffering from hyperandrogenic endocrinopathies such as congenital adrenal hyperplasia and Cushing's syndrome, may exhibit relative erythrocytosis (5, 6). The above historic findings underpin the androgens on stimulating bone morrow erythropoiesis.

## Testosterone (T) and Other Androgens

T, the principal circulating androgen in men, is a steroid hormone biosynthesized from cholesterol with its biological action mediated through binding and activation of androgen receptors in androgenic tissues after conversion, by 5α-reductase enzyme, to its more potent form dihydrotestosterone (DHT) (7). Since 1935, when the first T was isolated (8) different T preparations and routes were developed including several forms of the longer-acting T esters such T enanthate, T cypionate, and subsequently T undecanoate (9–11).

By 1970, several synthetic steroidal androgens were also available comprising groups of 17α-alkylated androgens, 1-methyl androgens, and nandrolone (12). As most of the oral 17α-alkylated androgens have hepatotoxic effect, they were not suitable as long term options for androgen replacement therapy (12). However, some of these androgens (such as danazol and stanozolol) along with other 1-methyl androgen (like methenolone) were used to treat aplastic anemia and anemia due to myelofibrosis (13–15).

Unlike T, nandrolone (19-nor testosterone) is metabolized by 5α-reductase to its much weaker metabolite 5α-dihydronandrolone (DHN), giving it a very high ratio of anabolic to androgenic activity (16). Due to this anabolic effect, it can be used in certain clinical indications such as severe burns, HIV-associated cachexia, and chronic obstructive lung diseases (17). Nevertheless, this anabolic potency unfortunately makes nandrolone and its derivatives (such as Trenbolone) an attractive substance to misuse for physique- and performance-enhancing purposes.

## Androgens and Erythropoiesis

Animal and human studies suggested a direct and indirect stimulatory effect of androgens on erythropoiesis, though the exact mechanism of such relation remains vaguely understood. Androgens administration results in an increase in the erythroid cell mass, the colony forming units for erythrocytes (CFU-E), and the production and secretion of Erythropoietin (EPO) (6) while androgen deprivation causes a reduction in red blood cell indices due to reduced proliferation of marrow erythroid precursors (18).

Androgens are converted to 17-keto-steroids capable of increasing the synthesis of mRNA in the nucleus causing differentiation of bone marrow cells from EPO-non-responsive to EPO responsive (6). Moreover, androgens enhances the glucose uptake resulting in glycolysis and gene transcription and mRNA synthesis in erythroid (19–21).

T may increase Hct by inhibiting secretion of hepcidin, the principal iron regulatory peptide, thereby leading to an increase in bioavailable iron (22) but may also enhance the incorporation of iron into the red blood cells (23) and improve red blood cells survival (24). Finally, the finding of raised insulin like growth factor 1 (IGF-1) levels in those receiving androgens suggested a potential link between androgens and an IGF-1 driven erythroid progenitor cells proliferation and differentiation (25, 26).

The effect of T on erythropoiesis is most pronounced during puberty, with prepubertal Hb being similar in boys and girls, but increases in boys after age 13 years in tandem with increasing T concentrations (6, 27). Boys with delayed puberty have Hb levels similar to those of prepubertal boys and girls, and treatment with T normalizes hemoglobin levels to those observed in the late male puberty (28, 29).

## Hypogonadism and Anemia of Chronic Kidney Disease (CKD)

Prior to the development of EPO therapy in the late 1980s, androgens were the only option for treating CKD-related anemia in men. Patients with CKD may have impairment of bone mineral density, muscle bulk, energy levels, quality of life, and sexual function, with adverse cardiovascular outcomes, especially in those with concurrent diabetes (30); while these features are likely multi-factorial in origin, they also occur during hypogonadism. Low T is prevalent among CKD patients and may contribute to renal anemia (31, 32). Up to two thirds of men on hemodialysis (HD) have serum T levels in the hypogonadal range, resulting from abnormalities at all levels in the hypothalamic-pituitary-testicular axis (33–36). Unpublished data from a cohort of 113 HD & 85 pre-dialysis (preD) stage 4 and 5 CKD patients reported a subnormal T levels were in 76% of pre-dialysis and 80% in HD males with a significant inverse correlation of Dα dose with total T (R −0.253; *p* < 0.01) and free T (−0.29; <0.01) (37).

T concentrations have been found to inversely correlate with all-cause and cardiovascular—related mortality, as well as with markers of inflammation in patients with dialysis dependent end stage renal disease (32, 38). Even in those with non-dialysis dependent kidney disease, low total T was associated with higher mortality (39). However, it remains unclear if T treatment in those with CKD-anemia and hypogonadism, could positively influence mortality in this high risk cohort.

The fact that hypogonadism is a well-established cause for anemia and reduced responsiveness to EPO in men with CKD may suggest a possible role for T therapy as an adjunct or an alternative to EPO in some men with CKD-related anemia (40). This is particularly relevant in some healthcare systems where CKD patients are unable to afford EPO therapy which might result in anemia requiring blood transfusion, especially while there is a lacking evidence that EPO improves morbidity or mortality in CKD (41).

The Cochrane systemic review for the use of androgen in treating anemia of CKD did not find sufficient evidence to confirm the benefit of androgen therapy in treating anemia of CKD (42). The Cochrane systemic review excluded 20 studies for either being non-randomized controlled trials, of <6 months duration, if treatment and control groups data were indistinct or if it was unknown if participants received any androgen therapy for the preceding 6 months. The review only included data from 8 studies with sample sizes ranging between 9 and 37 participants. Limitations of the included studies include lack of matching for hypogonadism at baseline, and the absence of titrating T doses to reach therapeutic levels. In a meta-analysis of 4 randomized controlled trial including men over the age of 50 years, nandrolone was non-inferior to EPO for the treatment of anemia of CKD, especially in developing countries where EPO might be unavailable (43).

It has been argued that androgen therapy may potentiate the effectiveness of EPO treatment and reduces the minimal EPO dose necessary to maintain satisfactory Hb level in patients undergoing HD (44). Additional benefits of T in patients with CKD may include improvement of sexual function (45), muscle mass, functional performance, quality of life and subsequently, could ameliorate frailty (46). However, these potential benefits require formal investigation to assess the clinical benefits vs. risk of therapy.

## Hypogonadism and Unexplained Anemia in the Elderly

Anemia in the elderly men may increase morbidity and mortality risk. In a retrospective study of men aged over 65 years admitted with acute myocardial infarction, lower Hct levels were associated with an increase in the 30-day mortality while treating the anemia improved the mortality rates (47). Similar observation of high mortality was reported in a cohort of anemia patients presenting with new onset heart failure (48).

Increasing age *per se* was previously thought to be a risk factor for developing anemia, since anemia prevalence dramatically rises after the age of 50 years and affects up to 20% of 85 year olds (49). Several causes for anemia in the elderly have been reported in the non-institutionalized United States population in the third National Health and Nutrition Examination Survey; nutritional factors, anemia of CKD, blood loss, bone marrow myelodysplasia were causative of anemia in around 2/3 of the population while in the remaining 1/3, anemia was unexplained (49, 50). Currently, the patho-physiological basis for “aging” anemia remains incompletely understood and the term “unexplained” anemia is still a common diagnosis in older men (51).

Hypogonadism and aging may both cause anemia, sarcopenia, and osteoporosis (52) with longitudinal and cross-sectional studies consistently showing a declining serum levels of T in aging men.

An analysis of the European male aging study (EMAS) data found that primary hypogonadism (PH) was strongly related to aging but not obesity, while secondary hypogonadism (SH) was linked to obesity, irrespective of age (53). In most aging men, “functional hypogonadism” is a reflection of the physiological burden of obesity, inflammation and accumulating comorbidities on the hypothalamus -pituitary-gonadal axis, however; it is unclear if this effect is adaptive (diverts resources from reproduction to survival), maladaptive (exacerbated disease-related frailty), or neutral. Indeed, the EMAS found that 1–2% of the general population of elderly men can show a clinical picture of PH or late onset hypogonadism (LoH), characterized by low T and raised gonadotrophins, attributed to age-related primary testicular failure (52, 54). Men with elevated serum follicle stimulating hormone (FSH) and normal levels of luteinizing hormone (LH) and T may have a reduced spermatogenesis with entirely normal testicular androgen production. In some men, a compensated dysregulation of gonadal function “compensated hypogonadism” is characterized by raised LH with normal T, reflecting a state of age-related health deterioration with potential progression to primary hypogonadism (55).

Whereas, organic secondary hypogonadism (low-normal LH+FSH) is biochemically indistinguishable from non-gonadal illness effect, PH can be easily diagnosable even in the acute (or chronic) disease settings, subject to the potential for the LH+FSH rise being blunted with intercurrent illness and/or medication (especially opiates).

In a retrospective study of 67 hospitalized elderly men (56), PH was found to be a surprisingly common cause of anemia among an unselected population of older male medical inpatients, 20.6% (7/34) of total cases with anemia and 43% (7/16) of those with otherwise unexplained anemia. Older men with PH and anemia therefore constitute a homogeneous group that can be reliably diagnosed and would thus represent an excellent potential cohort for examining the effects of T replacement therapy on haematopoiesis and other markers of frailty.

## Effect of T Therapy on Anemia

Studies have examined Hb changes in patients receiving T replacement therapy; some specifically trialing T therapy with the primary aim of improve anemia, with an accumulating body of evidence. In a randomized double blinded placebo-controlled study by Dhinsda et al., T therapy suppressed hepcidin with a marked increase in the Hb, EPO and expression of ferroportin and transferrin receptors in hypogonadal patients with type 2 diabetes (57). The National Institute of Health (NIH)-funded T Trials examined the effects of T therapy in hypogonadal men aged > 65 years (58). Using a double-blinded, placebo-controlled design, 12 months of daily T gel treatment increased Hb levels by at least 1.0 g/dL in ~52% of more men with hypogonadism and a known cause of anemia when compared with placebo. Furthermore, in the 64 older men with unexplained anemia; Hb improved by at least 1.0 g/dL in 54% of men vs. 15% in the placebo group. Nevertheless, androgen deficiency (AD) is typically overlooked in guidelines on the investigation of anemia (59).

It is important to note that most clinical guidelines recommend that Hct and prostate specific antigen (PSA) are assessed prior to initiating T therapy (60).

## Future Considerations

As life-expectancy increases, aging and frailty have become increasing health priorities worldwide. For instance, the number of people over 65 years of age is projected to reach 15–20% of the entire population by 2030 (61, 62). Unexplained anemia is common in older men, hypogonadism accounts for a proportion of these cases. Hypogonadism may negatively impact men's health and aggravate frailty, morbidity and mortality. Androgen therapy also has potential to treat anemia of CKD in hypogonadal men as an adjunct to EPO. Given the long-standing risks associated with T therapy, further studies are required to determine which men would most benefit functionally from T therapy without exposing them to unnecessary adverse effects or long-term complications.

## Author Contributions

AA-S drafted the structure of the article while AM and AA collected the relevant data as per AA-S guidance. AM and AA wrote the initial draft which was revised, edited, and expanded by AA-S. CJ supervised the manuscript's writing process and approved the final version.

### Conflict of Interest

CJ received an honorarium for debating the safety of testosterone therapy at a meeting organized by Society of Endocrinology sponsored by Besins Healthcare. The remaining authors declare that the research was conducted in the absence of any commercial or financial relationships that could be construed as a potential conflict of interest.
